# The relationship between music listening and subjective well-being: evidence from the Chinese General Social Survey (2010–2023)

**DOI:** 10.3389/fpsyg.2025.1716427

**Published:** 2025-12-11

**Authors:** Wei Zhu, Yuqin Ji, Song Liu

**Affiliations:** School of Economics and Management, Changzhou Institute of Technology, Changzhou, China

**Keywords:** music listening, music engagement, subjective well-being, happiness, health

## Abstract

**Background:**

Subjective well-being (SWB) has become a key indicator of quality of life, and growing evidence suggests that music listening can enhance SWB. However, most existing studies are based on Western contexts, with limited large-scale evidence from China.

**Methods:**

Using nationally representative data from the Chinese General Social Survey (CGSS, 2010–2023), supplemented with an online survey, this study examines the relationship between music listening and SWB. The CGSS employs standardized questionnaires and a stratified multistage probability sampling design; all analyses incorporate sampling weights and clustered standard errors. Ordered logistic regression models, robustness tests, heterogeneity analyses, and mechanism analyses were conducted to assess the association and its potential pathways.

**Results:**

Music listening is consistently and positively associated with SWB across all nine CGSS waves. This association remains robust to a series of tests. The strength of the association fluctuates slightly over time, weakening around 2021 but strengthening again in 2023. Heterogeneity analyses show that the association is stronger among women, older adults, the unemployed, individuals without a parter, and those with lower social or economic status. Mechanism analyses indicate that improvements in health may partially explain this relationship, and that individuals who perceive stronger health benefits from music tend to listen more frequently and spend more on music consumption.

**Conclusion:**

Music listening is positively associated with SWB in China, with health functioning as a potential pathway. Enhancing public awareness of music’s health benefits and expanding free or affordable access to music platforms may help promote wider engagement in music listening and support improvements in well-being.

## 1 Introduction

Music is a universal human activity for expressing and communicating. It exists in every society, varies more within than between societies (Mehr et al., 2019). The musical activities, including listening to music, singing, playing and creating, not only allow the expression of personal inner states and feelings, but also can bring many positive effects to those who engage in them (Welch et al., 2020). In conducting the literature review, we focused on empirical studies examining music listening, subjective well-being, and health-related mechanisms. Numerous studies concerning the benefits of musical activity have demonstrated that music can positively influence multiple dimensions of human life, including physical, emotional, cognitive, social, and educational aspects (North et al., 2000; Saarikallio and Erkkilä, 2007; Juslin and Västfjäll, 2008; Salimpoor et al., 2011; Dingle et al., 2013; Kappert et al., 2019; de Witte et al., 2020). Musical activities have been increasingly shown to exert a positive impact on people’s subjective well-being (SWB) (Laukka, 2007; Weinberg and Joseph, 2017; Daykin et al., 2018; Sheppard and Broughton, 2020; Dingle et al., 2021; Granot et al., 2021; Martínez-Castilla et al., 2021; Zhao et al., 2022; Viola et al., 2023; Rickard, 2025).

Listening to music is the most common and easily accessible form of music engagement in everyday life. Compared to music creating, playing and dancing, music listening requires fewer resources. The portability and flexibility of digital listening allow individuals to select music anytime and anywhere to meet emotional needs (Randall and Rickard, 2017). An increasing body of studies have demonstrated that music listening can enhance individuals’ SWB (Dingle et al., 2021; Viola et al., 2023). Schäfer et al. categorized the functions of music listening into three dimensions: regulating arousal and mood, achieving self-awareness, and expressing social relatedness (Schäfer et al., 2013). Groarke and Hogan developed a new measure to assess the functions of music listening (Groarke and Hogan, 2018). Boer and Abubakar revealed that the contribution of music listening within peer groups to well-being is evident across cultural contexts (Boer and Abubakar, 2014). The literature on music motivation is gradually becoming more substantial and nuanced. Morinville et al. confirmed that higher levels of self-determined motivation for listening to music predicted more SWB (Morinville et al., 2013). Rickard found that the psychological well-being effects of music listening depend on the underlying motivation (Rickard, 2025).

A substantial body of research has demonstrated the beneficial effects of music listening on SWB, yet empirical evidence from China remains scarce. Given that China is the world’s most populous country with a distinctive social and cultural background, it is both meaningful and necessary to investigate the relationship between music listening and SWB among Chinese residents. Moreover, most existing studies rely on relatively small samples, which limits the potential for heterogeneity analysis across demographic subgroups. In addition, the predominance of cross-sectional designs makes it difficult to capture temporal variations over time.

To address these research gaps, this study draws on nationally representative data from the Chinese General Social Survey (2010–2023) to examine the relationship between music listening and SWB. Specifically, it investigates: (i) whether a positive association exists between music listening and SWB among Chinese residents; (ii) how this association varies across demographic subgroups; and (iii) whether health may function as a potential pathway underlying this association.

## 2 Methods

### 2.1 Data sources

This study utilizes household survey data from nine waves of the Chinese General Social Survey (CGSS, 2010–2023). The CGSS is the earliest nationwide and continuous academic survey project in China and has been widely used in social science research (Shen et al., 2025). It employs a stratified, three-stage, probability-proportional-to-size (PPS) random sampling design to ensure representativeness across provinces, age groups, and socioeconomic backgrounds. Although the CGSS has been fielded since 2003, questions on music listening were introduced only in 2010. Therefore, this study uses data from the 2010–2023 waves. Supplementary Table S1 summarizes the sample flow across the nine CGSS waves. The initial dataset contains 96,417 observations, and after excluding cases with missing values for subjective well-being (SWB), music listening, or control variables, the final analytical sample includes 85,582 respondents. For the 2010–2018 waves, the loss rate due to item-level missingness was relatively low (1.5–3.4%), indicating no substantial missing-data problem. In 2021 and 2023, smaller effective sample sizes resulted from the CGSS assigning the SWB and music listening modules only to randomly selected subsamples.

To complement the CGSS data, this study conducted an online survey via wjx.cn (SoJump), a widely used survey platform in China, yielding 232 valid responses. The variable definition and descriptive statistics are presented in Supplementary Table S2.

### 2.2 Variable selection

#### 2.2.1 Dependent variable

The dependent variable is individuals’ SWB, measured by respondents’ answers to the question: “Overall, do you feel happy with your life?” Response options range from 1 = “very unhappy,” 2 = “relatively unhappy,” 3 = “neither happy or unhappy,” 4 = “relatively happy,” to 5 = “very happy.”

#### 2.2.2 Independent variable

The independent variable is the frequency of music listening, measured by responses to the question: “Have you often listened to music at home during your free time in the past year?” Response options are: 1 = “never,” 2 = “several times a year or less,” 3 = “several times a month,” 4 = “several times a week,” and 5 = “every day.”

#### 2.2.3 Control variables

Based on previous literature (Shen et al., 2025; Greb et al., 2018), three types of control variables are included at the individual, family, and region levels. The individual level controls include gender (“male” = 1, “female” = 0), age, ethnicity (“Han” = 1, “ethnic minority” = 0), religious belief (whether adhere to a religion: “yes” = 1, “no” = 0), years of education, marital status (“with a partner” = 1, “without a partner” = 0), social interaction (whether socialized or visited neighbors or friends during free time in the past year: “never” = 1, “rarely” = 2, “sometimes” = 3, “often” = 4, “very frequently” = 5), employment status (“employed” = 1, “unemployed” = 0), self-rated social status (ordered classification variables, assigned to 1–10), and personal socio-economic status (“lower class” = 1, “middle lower class” = 2, “middle class” = 3, “middle upper class” = 4, “upper class” = 5). The family level controls include family size (number of family members living together), and family economic status in the local area (“far below average” = 1, “below average” = 2, “average level” = 3, “above average” = 4, “far above average” = 5). The regional level controls include household location (“urban” = 1, “rural” = 0) and the province dummy variables.

#### 2.2.4 Descriptive statistics

**Figure 1:**
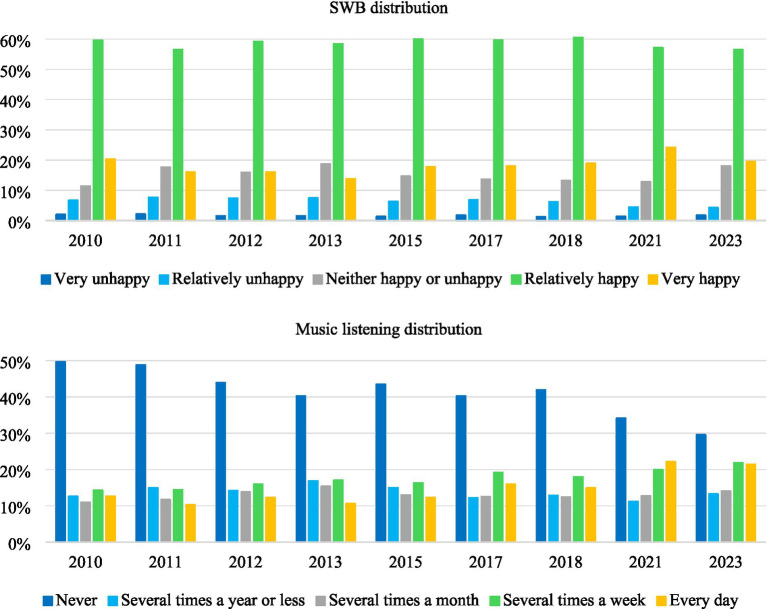
SWB and music listening distribution in the nine survey waves.

The descriptive statistics for all the variables are presented in Supplementary Table S3 and Figure 1 illustrates the distributions of SWB and music listening across the nine survey waves. The distribution of SWB shows that Chinese residents generally maintain a high level of happiness. The proportions of “relatively happy” and “very happy” respondents consistently exceed 75%. Meanwhile, the percentages of “unhappy” and “very unhappy” respondents remained below 10% throughout. The overall pattern suggests stable and predominantly positive well-being across the survey years.

The frequency of music listening shows a clear increase. The share of individuals who never listen to music declined sharply from nearly half of respondents in 2010 to below 30% in 2023. Meanwhile, the proportions of respondents listening “every day” or “several times a week” have more than doubled, reaching over 40% combined in recent years. Overall, these findings suggest that music has become a more regular and integral part of daily life.

Pearson correlation analysis reveals a consistently significant and positive relationship between music listening and SWB across all nine survey waves (Supplementary Figure S1). The correlation coefficients range from 0.073 to 0.136, all statistically significant at the 0.1% level, indicating a stable but modest positive association.

### 2.3 Model

Since the dependent variable is ordered categorical variable, the ordered logistic model is adopted to conduct empirical analysis. The model is shown in Equation 1:


$$\mathrm{SW}B_{i}=\alpha_{0}+\alpha_{1}\text{Musi}c_{i}+\sum_{2}^{j}\alpha_{j}X_{\mathrm{ji}}+\varepsilon_{i}$$ (1)

Where $\mathrm{SW}B_{i}$ indicates respondent *i*’s SWB; $\text{Musi}c_{i}$ refers the frequency of music listening; $X_{\mathrm{ji}}$ ’s are the control variables; $\alpha_{0}$ , $\alpha_{1}$ and $\alpha_{j}$ ’s are the estimated coefficients; and $\varepsilon_{i}$ is the random error term.

Given that the CGSS adopts a stratified, multistage sampling design, all analyses apply the sampling weights provided in each survey wave to ensure representativeness at the national level. To account for potential intra-cluster correlation arising from the sampling design, standard errors are clustered at the province level, thereby correcting for design effects and providing robust inference.

In order to test the robustness of the analysis results, this study also used propensity score matching, ordered probit, and ordinary least squares methods to analyze the relationship between music listening and SWB.

## 3 Results

### 3.1 Baseline regression

Table 1 presents the baseline results from the ordered logistic regression model using data from the CGSS 2010–2023. Across all nine survey waves, the odds ratios (ORs) for music listening range from 1.058 to 1.150, and are statistically significant at either the 0.1% (*p* < 0.001) or 1% (*p* < 0.01) level. These results indicate that individuals who listen to music more frequently tend to report higher levels of subjective well-being (SWB) than those who seldom or never listen to music, suggesting a consistently positive and stable association between music listening and SWB.

**Table 1:** Results of benchmark regression.

Variable	2010	2011	2012	2013	2015	2017	2018	2021	2023
Music listening	1.140*** (1.105–1.175)	1.150*** (1.102–1.200)	1.111*** (1.079–1.144)	1.130*** (1.095–1.165)	1.127*** (1.094–1.163)	1.114*** (1.084–1.145)	1.066*** (1.038–1.096)	1.058** (1.018–1.100)	1.087*** (1.048–1.128)
Gender	0.896** (0.831–0.967)	0.957 (0.856–1.071)	0.933+ (0.864–1.008)	0.924* (0.855–0.999)	0.932+ (0.860–1.010)	0.901** (0.837–0.970)	0.953 (0.885–1.026)	1.130* (1.007–1.099)	0.985 (0.883–1.099)
Age	1.010*** (1.007–1.014)	1.006** (1.002–1.011)	1.006*** (1.003–1.010)	1.006*** (1.003–1.010)	1.010*** (1.007–1.013)	1.013*** (1.010–1.015)	1.010*** (1.007–1.013)	1.012*** (1.007–1.016)	1.008*** (1.004–1.013)
Ethnicity	0.874+ (0.749–1.020)	0.625** (0.479–0.816)	0.908 (0.770–1.070)	0.713*** (0.605–0.841)	0.894 (0.754–1.061)	0.784** (0.665–0.923)	0.828* (0.700–0.978)	0.928 (0.707–1.218)	0.899 (0.717–1.129)
Religious belief	0.991 (0.880–1.117)	1.056 (0.880–1.270)	1.060 (0.945–1.188)	1.214** (1.068–1.381)	1.147* (1.010–1.302)	0.979 (0.862–1.110)	1.073 (0.948–1.215)	0.861 (0.683–1.086)	0.991 (0.810–1.214)
Years of education	1.017** (1.005–1.029)	0.983* (0.965–1.000)	1.010 (0.995–1.020)	0.998 (0.986–1.010)	1.009 (0.997–1.021)	1.019** (1.008–1.030)	1.000 (0.990–1.011)	1.020* (1.002–1.038)	1.028** (1.011–1.045)
Marital status	1.371*** (1.244–1.511)	1.209** (1.052–1.390)	1.352*** (1.227–1.489)	1.181** (1.071–1.303)	1.177*** (1.066–1.299)	1.251*** (1.143–1.369)	1.165*** (1.073–1.407)	1.229** (1.073–1.407)	1.147* (1.010–1.303)
Social interaction	1.058** (1.017–1.100)	1.034 (0.982–1.090)	1.103*** (1.062–1.146)	1.162*** (1.118–1.208)	1.118*** (1.076–1.161)	1.144*** (1.105–1.185)	1.111*** (1.073–1.151)	1.110*** (1.054–1.168)	1.102*** (1.050–1.157)
Employment status	0.935 (0.858–1.019)	0.971 (0.856–1.102)	0.822*** (0.753–0.897)	0.905* (0.830–0.988)	0.836*** (0.766–0.913)	0.850*** (0.783–0.922)	0.854*** (0.787–0.927)	0.838** (0.743–0.944)	0.851** (0.758–0.955)
Self-rated social status	1.275*** (1.243–1.307)	1.408*** (1.360–1.458)	1.281*** (1.249–1.313)	1.267*** (1.235–1.300)	1.273*** (1.238–1.309)	1.190*** (1.155–1.225)	1.161*** (1.127–1.196)	1.187*** (1.141–1.234)	1.122*** (1.083–1.162)
Personal socio-economic status	–	–	–	–	–	1.218*** (1.147–1.294)	1.336*** (1.256–1.421)	1.212*** (1.115–1.317)	1.410*** (1.303–1.527)
Family size	1.041** (1.012–1.071)	1.073** (1.030–1.117)	1.036* (1.007–1.065)	1.043** (1.014–1.074)	1.066*** (1.035–1.099)	1.063*** (1.035–1.093)	1.078*** (1.064–1.274)	1.078*** (1.044–1.114)	0.994 (0.963–1.026)
Family economic status	1.753*** (1.656–1.856)	1.491*** (1.381–1.610)	1.677*** (1.582–1.779)	1.780*** (1.676–1.906)	1.780*** (1.670–1.898)	1.523*** (1.430–1.620)	1.434*** (1.345–1.528)	1.524*** (1.396–1.665)	1.372*** (1.258–1.497)
Household location	1.101+ (0.987–1.227)	0.916 (0.787–1.065)	0.962 (0.865–1.070)	1.069 (0.963–1.187)	0.942 (0.847–1.049)	0.913+ (0.826–1.008)	1.030 (0.933–1.137)	1.103 (0.955–1.275)	1.066 (0.929–1.225)
Province	Yes	Yes	Yes	Yes	Yes	Yes	Yes	Yes	Yes
Pseudo *R*^2^	0.087	0.084	0.082	0.079	0.080	0.076	0.066	0.070	0.057
*N*	11,556	5,533	11,574	11,143	10,709	12,241	12,357	5,051	5,418

+*p* < 0.10; *p < 0.05; **p < 0.01, ***p < 0.001. Values outside parentheses are odds ratios (ORs). 95% CIs appear in parentheses; constant terms have been omitted. Yes means the variable is controlled.

During the earlier survey years (2010–2013), the estimated ORs exceed 1.13, implying a relatively stronger association between music listening and SWB. Although the ORs slightly decline after 2017, the relationship remains positive and robust across all years. This persistence suggests that the relationship between music listening and SWB is stable over time and not limited to a specific period. The modest year-to-year variation may reflect evolving listening habits, technological changes in music access, or generational differences in cultural participation.

It is important to emphasize that these results reflect associations rather than causal effects. Although individuals with higher SWB may also be more inclined to listen to music, the consistent pattern across multiple survey waves supports the predictive relevance of music listening in relation to SWB. In this sense, the frequency of music listening can be viewed as a reliable indicator of SWB levels within the Chinese population. Overall, the findings reveal a sustained and meaningful association between music listening and SWB.

### 3.2 Robustness tests

This study examines the robustness of the results in four ways. First, additional control variables were incorporated into the regression models to test the robustness and sensitivity of the estimated ORs for music listening, ensuring that the observed associations with SWB were not driven by omitted variable bias. Specifically, measures of social trust (Overall, do you agree that most people in society can be trusted? “strongly disagree” = 1, “disagree” = 2, “neither agree nor disagree” = 3, “agree” = 4, “strongly agree” = 5) and perceived social fairness (Overall, how fair do you think today’s society is? “completely unfair” = 1, “mostly unfair” = 2, “neither fair nor unfair” = 3, “mostly fair” = 4, “completely fair” = 5) were added to capture individual-level social and attitudinal characteristics. Several socio-economic factors were included—namely, household registration (hukou), personal annual income, household per capita income, number of properties owned, and car ownership. The ORs for music listening (Supplementary Table S4) remain highly significant and change only slightly after adding these variables, ranging from 1.058 to 1.149 compared with 1.058 to 1.150 in the baseline model. This consistency in magnitude and significance indicates that the positive association between music listening and SWB is stable and not sensitive to the inclusion of additional social and economic controls.

Second, music listening is transformed into a dummy variable: coded as 0 for respondents who choose “never” or “several times a year or less,” and 1 for others. The results (Supplementary Table S4) show that across all nine survey waves, the ORs for music listening range from 1.149 to 1.399, all statistically significant at the 0.1% level or 5% level, which is consistent with the baseline results.

Third, the sample was divided into two groups—frequent music listeners (who choose “every day” or “several times a week,” or “several times a month”) and infrequent listeners (who choose “never” or “several times a year or less”)—and the propensity score matching (PSM) method was employed to mitigate potential biases arising from non-treatment factors. The average treatment effects across all survey waves range from 0.043 to 0.140, with significance levels between 0.1 and 5% (Supplementary Table S4). These findings suggest that, after accounting for observable differences between frequent and infrequent listeners, individuals who listen to music more frequently tend to report higher levels of SWB.

Fourth, ordered probit (OP) and ordinary least squares (OLS) models were employed to examine whether the analytical results are sensitive to model specification. The consistency of the results (Supplementary Table S4) across different estimation techniques indicates that the observed relationship between music listening and SWB is not sensitive to model specification.

Overall, the findings remain stable and consistent across all four robustness tests. In all cases, the positive and statistically significant association between music listening and SWB persists, reinforcing the reliability and validity of the empirical results.

### 3.3 Heterogeneity analysis

**Figure 2:**
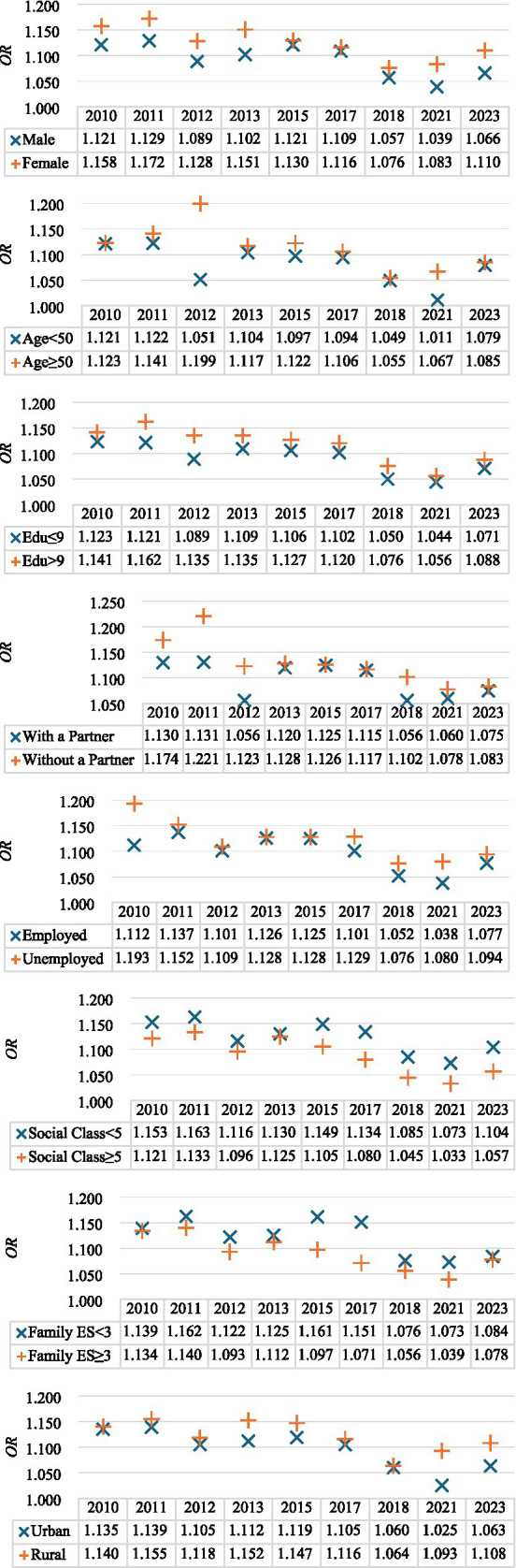
Results of heterogeneities analysis.

To gain deeper insight into the relationship between music listening and SWB, ordered logit regressions were conducted across subgroups defined by gender, age, years of education, marital status, employment status, self-rated social class, family economic status, and household location. The results reveal a consistent yet nuanced pattern across demographic groups (Figure 2).

Gender-stratified results indicate that the ORs for music listening are generally higher among females than males, suggesting that a stronger positive association between music listening and SWB among women. Across age groups, the association tends to be more pronounced among older individuals, possibly reflecting their deeper emotional engagement with music and its role in psychological regulation.

Similarly, the relationship appears stronger among respondents with higher education levels, underscoring the role of cultural capital in shaping the emotional and cognitive benefits of musical engagement. Individuals without a partner exhibit somewhat higher ORs than those with a partner, implying that music may serve as an alternative source of emotional support or companionship.

Employment status also plays a role: the ORs are slightly higher among unemployed individuals, suggesting that music listening may serve a compensatory function in sustaining well-being during periods of economic or social instability. Across social and economic dimensions, individuals with lower perceived social class or weaker family economic status exhibit stronger associations, implying that music may provide greater psychological comfort for relatively disadvantaged groups.

Furthermore, the positive association persists in both urban and rural areas, though it appears marginally stronger among rural residents—possibly reflecting the growing accessibility of digital music platforms in less urbanized regions.

Overall, these findings indicate that while the relationship between music listening and SWB is consistently positive, its strength varies across demographic and socioeconomic groups.

### 3.4 Mechanism analysis

To further explore the mechanisms underlying the positive association between music listening and SWB, an additional online survey was conducted to examine individuals’ motivations for listening to music. The results are summarized in Supplementary Figure S2. The most frequently reported reasons include “relaxing and relieving stress” (79.74%), “passing time” (53.45%), and “enjoying the pleasure brought by music” (53.02%). Other commonly mentioned motivations include “relieving anxiety or depression” (47.41%) and “assisting in sleep or improving sleep quality” (38.79%). Comparatively fewer respondents indicated listening to music for functional or social purposes, such as “assisting with learning, work, or exercise” (32.33%), or “appreciating the artistic value or cultural background of music” (26.72%). These findings suggest that improving mental and physical health represents an important motivation for music listening among respondents. At the same time, existing literature emphasize that health and functioning—including general health status, physical activity, sleep quality, and mental well-being—are among the most critical determinants of SWB (Das et al., 2020). Therefore, music listening may enhance SWB indirectly by promoting better health-related outcomes.

Therefore, this study employs ordered logit regressions to examine the association between music listening and health using data from the CGSS. Three health-related indicators—physical health, mental health, and health problems—are utilized to capture individuals’ overall health status. Physical health is measured by the question: “What do you think of your current physical health?” with five answers: 1 = “very unhealthy,” 2 = “relatively unhealthy,” 3 = “average,” 4 = “relatively healthy,” and 5 = “very healthy.” Mental health is measured by the question: “In the past 4 weeks, how often have you felt depressed?” with five answers: 1 = “always,” 2 = “often,” 3 = “sometimes,” 4 = “rarely,” and 5 = “never.” Health problems are assessed by the question: “In the past 4 weeks, how often has your work or other daily activities been affected by health problems?” using the same five answers as mental health.

The results are presented in Table 2. For physical health and health problems, the estimated ORs are consistently positive and highly significant across most survey waves. This indicates that individuals who listen to music more frequently tend to report better self-rated physical health and are less likely to experience disruptions in work or daily activities due to health issues. For mental health, the association remains positive in most years and statistically significant in several years, it appears weaker and less consistent than for physical health and health problems. Taken together, these results demonstrate that music listening is positively associated with multiple dimensions of health. These findings support the interpretation that improved health status may represent one of the mechanisms through which music listening predicts higher levels of SWB.

**Table 2:** The association between music listening and health.

Variable	2010	2011	2012	2013	2015	2017	2018	2021	2023
Physical health									
Music listening	1.049***	1.031	1.033*	1.088***	1.025+	1.054***	1.053***	1.006	1.054**
Controls	Yes	Yes	Yes	Yes	Yes	Yes	Yes	Yes	Yes
Pseudo *R*^2^	0.099	0.056	0.101	0.105	0.092	0.107	0.089	0.086	0.091
*N*	11,556	5,522	11,574	11,143	10,709	12,241	12,357	5,051	5,418
Mental health									
Music listening	1.035*	0.975	1.024+	1.070***	1.036*	1.018	1.006	0.990	1.045*
Controls	Yes	Yes	Yes	Yes	Yes	Yes	Yes	Yes	Yes
Pseudo *R*^2^	0.047	0.083	0.047	0.056	0.046	0.045	0.039	0.039	0.044
*N*	11,556	5,506	11,574	11,143	10,709	12,241	12,357	5,051	5,416
Health problems									
Music listening	1.086***	1.010	1.081***	1.132***	1.054***	1.047***	1.070***	1.024	1.043**
Controls	Yes	Yes	Yes	Yes	Yes	Yes	Yes	Yes	Yes
Pseudo *R*^2^	0.090	0.051	0.088	0.097	0.084	0.092	0.081	0.070	0.072
*N*	11,556	5,508	11,574	11,143	10,688	12,241	12,357	5,051	5,418

+*p* < 0.10; *p < 0.05; **p < 0.01, ***p < 0.001. Yes means the variable is controlled.

Given the established positive association between music listening and SWB, as well as its beneficial effects on health, an important question arises: how can public engagement in music be further promoted? In China, access to music is increasingly mediated through online platforms, many of which require paid subscriptions. Such economic barriers may limit participation in music-related activities and thereby constrain the potential public health and well-being benefits of music.

To explore this issue, this study employs online survey data to examine whether individuals’ perceptions of the health benefits of music listening influence their music-related behaviors. As shown in Supplementary Table S5, perceived physical health benefits of music—such as relieving pain, improving sleep quality, and enhancing exercise performance—are strongly and positively associated with music listening frequency, willingness to pay, and actual music consumption. Similarly, perceived mental health benefits also promote these behaviors, though with slightly weaker statistical significance. These findings suggest that recognizing music’s positive role in physical and mental health encourages individuals to engage in music listening more frequently and to invest more in music consumption.

## 4 Discussion

Using nationally representative data from nine waves of the China General Social Survey (CGSS, 2010–2023), this study provides robust and long-term evidence of a significant positive association between music listening and subjective well-being (SWB). This finding aligns with prior international studies (Dingle et al., 2021; Granot et al., 2021; Viola et al., 2023) and extends the evidence to a broader temporal and cultural context. The relationship remains consistently positive across all survey years and demographic subgroups, and is robust to multiple estimation techniques and the inclusion of extensive social and economic control variables.

From a temporal perspective, the odds ratios (ORs) of music listening are relatively high in the early years (2010–2017), indicating that music listening was strongly associated with SWB during China’s rapid social and cultural transformation. After 2017, the strength of this relationship declined slightly, reaching its lowest level in 2021. A plausible explanation lies in the social and psychological impacts of the COVID-19 pandemic. During the 2021 survey period, widespread lockdowns and mobility restrictions gave people more leisure time, allowing them to engage not only in music listening but also in various alternative recreational activities (Martínez-Castilla et al., 2021; Cabedo-Mas et al., 2021). By 2023, however, the relationship between music listening and SWB strengthened again. Although pandemic restrictions had been lifted, the lingering health risks and economic slowdown continued to generate social stress and uncertainty (Xiao et al., 2023; Wu et al., 2023). Under such circumstances, music listening may have regained importance as a coping mechanism for emotional regulation and stress relief, amplifying its association with SWB.

The heterogeneity analysis reveals that the positive association between music listening and SWB is stronger among women, older and more educated individuals, the unemployed, individuals without a partner, and those with lower social or economic status. This pattern suggests that music may play a more salient emotional or compensatory role within these population groups. For older adults, music may provide companionship and cognitive stimulation, thereby helping to mitigate the risks of social isolation (Hays and Minichiello, 2005). Single individuals may rely on music as an emotional substitute for intimate relationships (Lonsdale and North, 2011). Those with limited socio-economic resources may benefit from music as an affordable and accessible form of leisure (Boer and Fischer, 2012). Collectively, these findings suggest that music listening is particularly valuable for groups experiencing greater social or material vulnerability.

The mechanism analysis suggests that improvements in health may represent an important pathway through which music listening predicts higher levels of SWB. By promoting relaxation, reducing stress, and supporting better physical and emotional balance, music listening appears to enhance individuals’ perceived health status and thereby reinforce their overall well-being (Dingle et al., 2021; Viola et al., 2023). The comparatively weaker association between music listening and mental health may indicate that emotional motivations for listening—combined with variations in listeners’ initial mood states—can sometimes place individuals at risk of less desirable psychological outcomes (Rickard, 2025; Randall and Rickard, 2017).

Furthermore, results from the online survey indicate that individuals’ perceptions of the health benefits of music listening significantly influence their music-related behaviors, consistent with prior research (Cabedo-Mas et al., 2021). Specifically, perceiving music as beneficial for physical and mental health positively predicts listening frequency, willingness to pay for music listening, and music listening-related expenditure.

From a policy perspective, this study highlights that music listening is positively associated with both SWB and health, and that individuals’ recognition of music’s health benefits significantly increases their listening frequency and willingness to pay. Therefore, policymakers should view music engagement not merely as a cultural activity but also as a form of health promotion and public well-being enhancement. Governments and cultural institutions could strengthen public awareness of the physical and mental health benefits of music through campaigns, educational programs, and media initiatives. Providing affordable or free access to music—through publicly funded platforms, community radio, or digital streaming services—could reduce economic barriers and encourage broader participation, particularly among low-income and rural populations. Additionally, supporting community-based and participatory music activities, such as local concerts or singing groups, may foster social interaction and collective well-being. By integrating cultural policy with public health objectives, such measures can expand access to music, promote healthy lifestyles, and enhance overall happiness in society.

Several limitations of this study should be acknowledged. First, the measurement of music listening is restricted to the reported “frequency of listening to music at home during free time.” This indicator does not capture other important dimensions such as listening context, musical genre, or emotional engagement. As a result, individuals who primarily listen to music in other settings (e.g., during commutes, at work, or while exercising) may be misclassified as low-frequency listeners, which could affect construct validity and underestimate the broader influence of music on well-being. Second, missing data on key variables (SWB and music listening) were handled using listwise deletion. Although the overall sample loss rate was modest, listwise deletion still relies on the assumption that missingness is completely at random, which may introduce some degree of selection bias. Finally, the present design cannot fully address potential reverse causality: individuals with higher SWB may also be more inclined to engage in music listening. While the analyses identify robust associations, causal inference remains limited. Future research employing longitudinal or cross-lagged panel approaches would be valuable in disentangling the directionality of this relationship.

## Data Availability

Publicly available datasets were analyzed in this study. This data can be found at: https://www.cnsda.org/.
