# Supplementary material for: The relationship between music listening and subjective well-being: evidence from the Chinese General Social Survey (2010–2023)
Source: Front Psychol. 2025 Dec 11;16:1716427. doi: 10.3389/fpsyg.2025.1716427 (PMC12739865; doi:10.3389/fpsyg.2025.1716427)
Supplement: Supplementary file 1 [file Table_1.docx]

Supplementary material

TABLE S1 Sample sizes and missing-data patterns across CGSS waves (2010–2023).

| Year | 2010 | 2011 | 2012 | 2013 | 2015 | 2017 | 2018 | 2021 | 2023 |
| --- | --- | --- | --- | --- | --- | --- | --- | --- | --- |
| Total sample size | 11,783 | 5,620 | 11,765 | 11,438 | 10,968 | 12,582 | 12,787 | 8,148 | 11,326 |
| Sample reporting SWB | 11,767 | 5,614 | 11,724 | 11,380 | 10,953 | 12,561 | 12,772 | 5,448 | 11,307 |
| Sample reporting music listening | 11,694 | 5,583 | 11,732 | 11,391 | 10,944 | 12,570 | 12,770 | 8,124 | 7,059 |
| Sample with complete data | 11,556 | 5,533 | 11,574 | 11,143 | 10,709 | 12,241 | 12,357 | 5,051 | 5,418 |
| Sample loss rate | 1.93% | 1.55% | 1.62% | 2.58% | 2.36% | 2.71% | 3.36% |  |  |

1. “Sample with complete data” refers to respondents with non-missing values on SWB, music listening, and all covariates used in the baseline regression models.
2. In 2021 and 2023, the CGSS randomly assigned certain survey modules to subsamples. As a result, only a portion of respondents were asked the SWB or music-listening questions. Because missingness in these two years was due to the survey design rather than item nonresponse, sample loss rates were not calculated for 2021 and 2023.

TABLE S2 Variable definition and descriptive statistics for online survey.

| Variable | Description | Mean | Std. Dev. | Min | Max |
| --- | --- | --- | --- | --- | --- |
| Music listening | Frequency of music listening in the past year: Never = 1, Several times a year or less = 2, Several times a month = 3, Several times a week = 4, Every day = 5 | 4.095 | 0.822 | 1 | 5 |
| WTP | Willingness to pay for music listening | 0.767 | 0.424 | 0 | 1 |
| Music consumption | Consumption expenditure of music listening (Yuan) | 2.552 | 1.131 | 1 | 5 |
| Physical health effect | Physical health effect of music listening: Strongly disagree = 1, Somewhat disagree = 2, Neither agree or disagree = 3, Somewhat agree = 4, Strongly agree = 5 | 3.944 | 0.751 | 1 | 5 |
| Mental health effect | Mental health effect of music listening: Strongly disagree = 1, Somewhat disagree = 2, Neither agree or disagree = 3, Somewhat agree = 4, Strongly agree = 5 | 4.211 | 0.711 | 2 | 5 |
| Gender | Male = 1, Female = 0 | 0.401 | 0.491 | 0 | 1 |
| Age | Age (year): Below 18 =1, 18 to 24 = 2, 25 to 29 = 3, 30 to 39 = 4, 40 to 49 = 5, 50 to 59 = 6, 60 and over = 7 | 3.573 | 1.062 | 1 | 7 |
| Education | Years of education | 15.802 | 1.137 | 11 | 18 |
| Marital status | With a partner = 1, Without a partner = 0 | 0.672 | 0.470 | 0 | 1 |
| Income | Monthly income (Yuan): Below 2000 = 1, 2000 to 2999 = 2, 3000 to 4999 =3, 5000 to 7999 = 4, 8000 to 9999 = 5, 10000 to 14999 = 6, 15000 and over =7 | 4.315 | 1.698 | 1 | 7 |
| Employment | Employed = 1, Unemployed = 0 | 0.879 | 0.326 | 0 | 1 |
| City level | Development level of the city | 2.728 | 1.065 | 1 | 4 |

TABLE S3 Descriptive statistics of the variables across CGSS waves (2010–2023).

| Variable | 2010 | 2011 | 2012 | 2013 | 2015 | 2017 | 2018 | 2021 | 2023 |
| --- | --- | --- | --- | --- | --- | --- | --- | --- | --- |
| SWB | 3.766  (0.883) | 3.897  (0.867) | 3.808  (0.845) | 3.755  (0.839) | 3.867  (0.821) | 3.855  (0.851) | 3.898  (0.816) | 3.983  (0.820) | 3.895  (0.834) |
| Music listening | 2.224  (1.432) | 2.275  (1.494) | 2.383  (1.472) | 2.408  (1.422) | 2.389  (1.473) | 2.583  (1.544) | 2.511  (1.530) | 2.842  (1.592) | 2.923  (1.543) |
| Gender | 0.482  (0.500) | 0.457  (0.498) | 0.512  (0.500) | 0.503  (0.500) | 0.468  (0.499) | 0.472  (0.499) | 0.467  (0.499) | 0.456  (0.498) | 0.495  (0.500) |
| Age | 47.303  (15.679) | 48.160  (16.038) | 48.907  (16.256) | 48.597  (16.388) | 50.397  (16.898) | 51.009  (16.864) | 51.830  (16.948) | 51.812  (17.633) | 51.867  (16.791) |
| Ethnicity | 0.905  (0.293) | 0.945  (0.229) | 0.914  (0.281) | 0.914  (0.280) | 0.921  (0.270) | 0.925  (0.264) | 0.927  (0.260) | 0.925  (0.264) | 0.913  (0.281) |
| Religious belief | 0.130  (0.336) | 0.111  (0.314) | 0.147  (0.354) | 0.112  (0.315) | 0.121  (0.326) | 0.104  (0.306) | 0.107  (0.309) | 0.076  (0.265) | 0.089  (0.284) |
| Years of education | 8.573  (4.505) | 8.405  (4.450) | 8.562  (4.540) | 8.653  (4.565) | 8.567  (4.634) | 8.964  (4.687) | 8.718  (4.841) | 9.164  (4.607) | 9.898  (4.654) |
| Marital status | 0.805  (0.396) | 0.795  (0.404) | 0.798  (0.402) | 0.791  (0.407) | 0.781  (0.413) | 0.767  (0.423) | 0.765  (0.424) | 0.729  (0.445) | 0.725  (0.447) |
| Social interaction | 2.634  (1.018) | 2.739  (1.086) | 2.741  (1.061) | 2.854  (1.004) | 2.806  (1.047) | 2.723  (1.057) | 2.720  (1.059) | 2.651  (1.130) | 2.648  (1.135) |
| Employment status | 0.639  (0.480) | 0.651  (0.477) | 0.638  (0.481) | 0.626  (0.484) | 0.572  (0.495) | 0.559  (0.497) | 0.539  (0.498) | 0.502  (0.500) | 0.513  (0.500) |
| Self-rated social status | 4.062  (1.731) | 4.146  (1.799) | 4.171  (1.706) | 4.314  (1.680) | 4.316  (1.638) | 4.132  (1.706) | 4.226  (1.675) | 4.284  (1.851) | 4.285  (1.881) |
| Personal socio-economic status |  |  |  |  |  | 2.218  (0.874) | 2.297  (0.862) | 2.279  (0.903) | 2.282  (0.888) |
| Family size | 2.934  (1.389) | 2.939  (1.411) | 3.056  (1.410) | 3.092  (1.416) | 2.892  (1.412) | 2.804  (1.430) | 2.808  (1.443) | 3.448  (1.886) | 3.634  (2.125) |
| Family economic status | 2.611  (0.766) | 2.552  (0.790) | 2.624  (0.731) | 2.684  (0.681) | 2.652  (0.717) | 2.547  (0.756) | 2.576  (0.730) | 2.598  (0.775) | 2.608  (0.763) |
| Household location | 0.613  (0.487) | 0.575  (0.494) | 0.607  (0.489) | 0.608  (0.488) | 0.590  (0.492) | 0.639  (0.480) | 0.712  (0.453) | 0.557  (0.497) | 0.618  (0.486) |
| N | 11,556 | 5,533 | 11,574 | 11,143 | 10,709 | 12,241 | 12,357 | 5,051 | 5,418 |

Values outside parentheses indicate means, and values in parentheses indicate standard deviations.

FIGURE S1 Correlation coefficients between music listening and SWB.

*** p < 0.001.

TABLE S4 Results of robustness tests.

| Variable | 2010 | 2011 | 2012 | 2013 | 2015 | 2017 | 2018 | 2021 | 2023 |
| --- | --- | --- | --- | --- | --- | --- | --- | --- | --- |
| With additional controls | | | | | | | | | |
| Music listening | 1.139*** | 1.149*** | 1.108*** | 1.126*** | 1.127*** | 1.113*** | 1.065*** | 1.058** | 1.082*** |
| Controls | Yes | Yes | Yes | Yes | Yes | Yes | Yes | Yes | Yes |
| Pseudo *R*^2^ | 0.087 | 0.089 | 0.083 | 0.079 | 0.081 | 0.077 | 0.068 | 0.070 | 0.095 |
| N | 11,455 | 5,533 | 11,533 | 11,143 | 10,547 | 12,132 | 12,209 | 5,000 | 5,232 |
| Music dummy | | | | | | | | | |
| Music listening | 1.399*** | 1.417*** | 1.250*** | 1.270*** | 1.369*** | 1.301*** | 1.160*** | 1.149* | 1.205*** |
| Controls | Yes | Yes | Yes | Yes | Yes | Yes | Yes | Yes | Yes |
| Pseudo *R*^2^ | 0.087 | 0.083 | 0.081 | 0.078 | 0.079 | 0.076 | 0.066 | 0.069 | 0.056 |
| N | 11,556 | 5,533 | 11,574 | 11,192 | 10,709 | 12,241 | 12,357 | 5,051 | 5,396 |
| PSM | | | | | | | | | |
| Music listening | 0.125*** | 0.140*** | 0.062** | 0.080*** | 0.104*** | 0.105*** | 0.064*** | 0.043* | 0.073** |
| Controls | Yes | Yes | Yes | Yes | Yes | Yes | Yes | Yes | Yes |
| N | 10,361 | 4,895 | 10,032 | 9,692 | 9,298 | 10,014 | 10,426 | 3,797 | 4,413 |
| Order Probit | | | | | | | | | |
| Music listening | 0.727*** | 0.079*** | 0.056*** | 0.062*** | 0.064*** | 0.061*** | 0.036*** | 0.031** | 0.042*** |
| Controls | Yes | Yes | Yes | Yes | Yes | Yes | Yes | Yes | Yes |
| Pseudo *R*^2^ | 0.086 | 0.083 | 0.080 | 0.076 | 0.079 | 0.076 | 0.066 | 0.069 | 0.057 |
| N | 11,556 | 5,533 | 11,574 | 11,192 | 10,709 | 12,241 | 12,357 | 5,051 | 5,396 |
| OLS | | | | | | | | | |
| Music listening | 0.047*** | 0.050*** | 0.034*** | 0.039*** | 0.042*** | 0.042*** | 0.022*** | 0.022** | 0.030*** |
| Controls | Yes | Yes | Yes | Yes | Yes | Yes | Yes | Yes | Yes |
| *R*^2^ | 0.795 | 0.793 | 0.773 | 0.769 | 0.748 | 0.778 | 0.757 | 0.757 | 0.791 |
| N | 11,556 | 5,533 | 11,574 | 11,192 | 10,709 | 12,241 | 12,357 | 5,051 | 5,396 |

*p < 0.05; **p < 0.01, ***p < 0.001. Yes means the variable is controlled.

FIGURE S2 Motivations for listening to music.

TABLE S5 Impact of perception on music-related behaviors.

| Variable | Music listening | | WTP | | Music consumption | |
| --- | --- | --- | --- | --- | --- | --- |
| Physical health effect | 0.736** |  | 0.703** |  | 0.583** |  |
|  | (0.239) |  | (0.244) |  | (0.171) |  |
| Mental health effect |  | 0.457+ |  | 0.404+ |  | 0.228+ |
|  |  | (0.249) |  | (0.212) |  | (0.117) |
| Controls | Yes | Yes | Yes | Yes | Yes | Yes |
| Pseudo *R*^2^ | 0.061 | 0.043 | 0.115 | 0.086 | 0.065 | 0.051 |
| N | 232 | 232 | 232 | 232 | 232 | 232 |

+ p < 0.10; *p < 0.05; **p < 0.01, ***p < 0.001.
